# A Clinical Tool to Predict Low Serum Selenium in Patients with Worsening Heart Failure

**DOI:** 10.3390/nu12092541

**Published:** 2020-08-21

**Authors:** Ali A. Al-Mubarak, Niels Grote Beverborg, Stefan D. Anker, Nilesh J. Samani, Kenneth Dickstein, Gerasimos Filippatos, Dirk Jan van Veldhuisen, Adriaan A. Voors, Nils Bomer, Peter van der Meer

**Affiliations:** 1 Department of Cardiology, University Medical Center Groningen, University of Groningen, 9700 RB Groningen, The Netherlands; a.al.mubarak@umcg.nl (A.A.A.-M.); n.grote.beverborg@umcg.nl (N.G.B.); d.j.van.veldhuisen@umcg.nl (D.J.v.V.); a.a.voors@umcg.nl (A.A.V.); 2 Innovative Clinical Trials, Department of Cardiology and Pneumology, University Medical Centre Göttingen (UMG), 37075 Göttingen, Germany; s.anker@cachexia.de; 3 Department of Cardiovascular Sciences, University of Leicester, Leicester LE39QP, UK; njs@leicester.ac.uk; 4 NIHR Leicester Biomedical Research Centre, Glenfield Hospital, Leicester LE39QP, UK; 5 Stavanger University Hospital, 4011 Stavanger, Norway; trout@online.no; 6 Department of Clinical Science, University of Bergen, 5007 Bergen, Norway; 7 Department of Cardiology, Heart Failure Unit, Athens University Hospital Attikon, 124 06 Athens, Greece; geros@otenet.gr

**Keywords:** selenium, malnutrition, heart failure, albumin, iron deficiency

## Abstract

Selenium is an essential micronutrient, and a low selenium concentration (<100 µg/L) is associated with a poorer quality of life and exercise capacity, and an impaired prognosis in patients with worsening heart failure. Measuring selenium concentrations routinely is laborious and costly, and although its clinical utility is yet to be proven, an easy implemented model to predict selenium status is desirable. A stepwise multivariable logistic regression analysis was performed using routinely measured clinical factors. Low selenium was independently predicted by: older age, lower serum albumin, higher N-terminal pro-B-type natriuretic peptide levels, worse kidney function, and the presence of orthopnea and iron deficiency. A 10-points risk-model was developed, and a score of ≥6 points identified >80% of patients with low selenium (sensitivity of 44%, specificity of 80%). Given that selenium and iron overlap in their physiological roles, we evaluated the shared determinants and prognostic associates. Both deficiencies shared similar clinical characteristics, including the model risk factors and, in addition, a low protein intake and high levels of C-reactive protein. Low selenium was associated with a similar or worse prognosis compared to iron deficiency. In conclusion, although it is difficult to exclude low selenium based on clinical characteristics alone, we provide a prediction tool which identifies heart failure patients at higher risk of having a low selenium status.

## 1. Introduction

Heart failure (HF) is a clinical syndrome resulting from impaired ventricular filling or the ejection of blood. HF accounts for substantial morbidity and mortality, with increasing prevalence globally [1]. Various factors play a causative role in developing HF, including myocardial infarction, hypertension, diabetes, and valvular heart diseases [1]. Moreover, in the last decade micronutrient deficiencies have been implicated in the development and progression of HF [2]. An aberrant equilibrium of minerals and trace elements (for example, iron, iodine, and zinc) in patients’ circulation is closely associated with the development and progression of HF [3,4]. Up to 50% of patients with HF suffer from some form of malnutrition, such as micronutrient insufficiencies [5,6]. A deficiency in the trace element iron was associated with decreased cardiomyocyte contractility and increased morbidity and mortality [7,8,9], whereas treatment with intravenous iron has been shown to relieve symptoms [10,11,12]. Phase III clinical trials with the aim of improving patient prognosis are currently being conducted (NCT02937454) [13].

Selenium (Se) is an essential micronutrient which has been less intensively studied though previous studies, suggesting that very severe deficiency in humans is associated with cardiomyopathy (Keshan disease) [3,14]. Recently, we showed that approximately 25% of patients with worsening HF have a serum Se < 70 μg/L, and that this was associated with a poorer quality of life, poor exercise capacity, and a worse prognosis [15]. Furthermore, a serum Se concentration of 70–100 μg/L appeared to have similar adverse associations, suggesting that values < 100 μg/L might be considered insufficient for the physiological requirement and thus abnormal [16,17]. The supplementation of Se is relatively simple and safe, but its clinical utility has yet to be proven. However, since Se is not measured routinely in clinics due to the labor intensity and subsequent high costs, identifying patients with a low Se is challenging. Furthermore, Se and iron share several physiological functions, such as redox homeostasis [3,9], angiogenesis [18], and immune response [3,9]. Nevertheless, the effects of the potential shared risk factors and interactions between both deficiencies on the prognosis of HF patients are not known.

In this study, we used the clinical predictors of a low serum Se concentration (<100 μg/L) in patients with worsening HF to develop a model capable of predicting the presence of a low Se level. Such a model will provide insights of the clinical factors related to Se and could improve the selection of patients that might benefit from Se supplementation, although this needs to be evaluated in clinical trials. In addition, we studied the levels of both Se and iron in association with the identified clinical predictors. Finally, we investigated the associations of Se and iron with prognosis independent from each other in established risk-models.

## 2. Materials and Methods

### 2.1. Study Population

We used data from the BIOSTAT-CHF index cohort (a systems BIOlogy Study to TAilored Treatment in Chronic Heart Failure), which has been described previously [19,20]. In short, BIOSTAT-CHF is a European cohort consisting originally of 2516 patients from 69 centers in 11 European countries. The included patients had to be 18 years old or older, with symptoms of new-onset or worsening HF. Their cardiac function has to be characterized by at least one of the following: a left ventricular ejection fraction (LVEF) of ≤40%, a plasma B-type natriuretic peptide (BNP) of >400 pg/mL, or a plasma N-terminal pro-B-type natriuretic peptide (NT-proBNP) of >2000 pg/mL. In addition, patients could only be included if they were sub-optimally treated for HF at inclusion (i.e., ≤50% of angiotensin-converting enzyme inhibitors, angiotensin receptor blockers, and/or beta-blockers). Patients with sepsis, acute myocarditis, or monogenic cardiomyopathy were excluded. The study was conducted in consensus with the Declaration of Helsinki. Informed consent was obtained from all the participants prior to any study-related activities.

### 2.2. Laboratory Measurements and Related Definitions

Blood samples were collected during the index hospitalization or at the outpatient clinic. Approximately 80 mL of blood plasma and serum were collected and stored at −80 °C. The serum Se was measured using the validated inductively coupled plasma mass spectrometry (ICP-MS) method, as described previously [15]. A low serum Se concentration was set at serum levels of below 100 μg/L based on previous observations in patients with worsening HF [15]. The serum NT-proBNP concentrations were measured using an immunoassay based on electrochemiluminescence (Elecsys, Roche Diagnostics, Mannheim, Germany). Kidney function was calculated using the Modification of Diet in Renal Disease (MDRD) equation. Anemia was defined according to the WHO standards (hemoglobin level < 12 g/dL in women and <13 g/dL in men). The iron-related parameters were measured as described previously by van der Wal et al. [9], and iron deficiency (ID) was defined as a transferrin saturation (TSAT) < 20% [7,21]. HF with reduced ejection fraction (HFrEF) was defined as a left ventricular ejection fraction < 40%.

### 2.3. Statistical Analysis

#### 2.3.1. Baseline Characteristics

All the statistical analyses were performed using Stata v.16 SE and Weka v.3.6.15. The patients were divided into two groups based on their Se status (low Se (<100 μg/L) vs. normal Se (≥100 μg/L)). Variables with a normal distribution were presented as a mean (standard deviation), while continuous variables with a non-normal distribution were presented as a median (interquartile range), and categorical variables were presented as a count (percentage). The baseline characteristics were analyzed using a t-test for continuous variables with a normal distribution, the Mann–Whitney U test for continuous variables with skewed data, and the Chi-square test for categorical variables.

#### 2.3.2. Clinical Predictors of Low Se

To identify independent predictors of low Se, a stepwise multivariable logistic regression analysis was performed. The potential determinants that can enter this analysis had to fulfill the following criteria: (i) a significant difference between the stratified groups in the baseline table, (ii) the potential predictor had to be used/measured/obtained routinely in the clinical practice, and (iii) the potential determinant should not have missing values > 15%. If two biologically similar variables fulfilled these criteria (i.e., hemoglobin and anemia), only one variable was included in the analysis. The variables fulfilling these criteria are summarized in Table A1. After the stepwise regression, a multivariable logistic regression was performed on the selected variables to accurately establish their association with Se in the entire cohort. As a sensitivity analysis, a multivariable Lasso logistic regression model as well as a bootstrap analysis were performed, using the “Lasso” and “swboot” packages in Stata. The multicollinearity of the final model was checked by calculating the variance inflation factor, the values of which should not exceed a maximum of 10 [22].

#### 2.3.3. Prediction Model

The optimal cutoff values for the independent predictors were determined using the unsupervised discretization tool in Weka [23]. Three not necessarily equal clusters were formed per continuous variable, the cutoff values of which were used to establish a risk-model. Two points were given to the values that favor low Se concentration the most, 1 point to the middle values, and 0 point to the remaining group. After determining the number of patients matching each score, the Odds Ratio’s (OR) of each score having a low Se were calculated. The test characteristics (i.e., sensitivity, specificity) were calculated in order to provide insights on the performance of the risk-model.

#### 2.3.4. Comparing Determinants and Prognosis of Low Se and ID

In order to visualize the prevalence of ID in comparison to low Se with regard to the identified predictors, restricted cubic splines (five knots for all variables) were constructed. The prevalence of anemia was also assessed in these analyses as a reference. The influence of low Se and ID on the outcome was further assessed using a Cox proportional hazard regression analysis. Both low Se and ID were added together in the models to assess the independency and additionally corrected for the BIOSTAT-CHF risk-models that predict the 2-year combined endpoint of HF hospitalizations and mortality and the 2-year all-cause mortality alone [20].

## 3. Results

### 3.1. Patient Characteristics

The mean Se concentration in the studied cohort of 2328 HF patients was 89.1 (24.8) μg/mL, with 1633 (70.1%) of patients having Se values < 100 μg/L. The differences between patients with a low Se and a normal Se were largely similar to those previously reported for patients with a Se deficiency (<70 μg/L) [15]. Patients with a low Se were significantly older (70.1 [±11.9] years vs. 66.0 [±11.8], *p* < 0.001), were more likely to be female (28.0% vs. 21.6%, *p* < 0.001), had worse symptoms (39.6% NYHA class III/IV vs. 30.1%, *p* < 0.001), were more likely to have a left ventricular ejection fraction >40% (21.3% vs. 12.9%, *p* < 0.001), and had a higher prevalence of renal insufficiency (estimated Glomerular Filtration Rate (eGFR) < 60 mL/min) (48.0% vs. 41.4%, *p* < 0.001). Furthermore, low Se was associated with a pro-inflammatory profile (median IL6; 5.8 [3.2–11.4] vs. 3.9 [2.1–7.4] μg/L, *p* < 0.001) and median C-reactive protein (CRP) (14,151.5 [6760.5–27,734.3] vs. 11,091.9 [4127.5–22,689.7] mg/L, *p* < 0.001), as well as higher cardiac biomarkers. In addition, ID was present in 1436 (62.0%) patients, while anemia was present in 772 (36.3%) patients (Table A2). The prevalence of both ID (66.4% vs. 51.7%, *p* < 0.001) and anemia (39.2% vs. 28.9%, *p* < 0.001) was higher in patients with low Se.

### 3.2. The Clinical Predictors and Diagnostic Accuracy of the Risk-Model

Older age, lower serum albumin, higher NT-proBNP levels, worse kidney function, and the presence of orthopnea and ID were independent predictors for low Se status (Table 1, c-statistic 0.6923). These determinants remained highly selective in the additional sensitivity analyses using bootstrapping and Lasso penalized regression (Table A3 and Table A4). No multicollinearity was detected (variance inflation factors: 1.05–1.27). Although there were some variations between the selenium levels among the included countries, the geographical location did not remain statistically significant as a predictor in the stepwise multivariable logistic regression analysis (Table A5).

**Figure 1:**
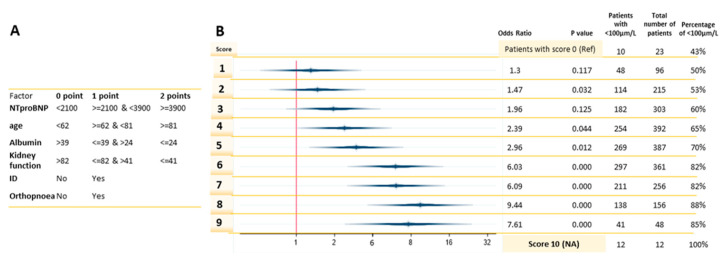
A risk-model using clinical variables to predict low Se. (**A**) Point distribution and cutoffs based on the unsupervised discretization. (**B**) Logistic regression of the risk-model with low selenium concentrations (<100 µg/L) as the outcome variable. Score 0 was the reference score.

Unsupervised discretization resulted in clusters with different densities per group (Table A6). NT-proBNP values ≥ 3900 ng/L, an age of 81 years-old or older, albumin levels ≤ 24 g/L, or a kidney function ≤ 41 mL/min/1.73 m^2^ qualified patients to receive two points for each variable (Figure 1A). The percentage of patients with a low selenium concentration increases with higher scores, ranging from 43% of the total patients who scored 0 points to 100% who scored 10 points (Figure 1B). A score of ≥6 points identified > 80% of patients with low selenium (sensitivity of 44%, specificity of 80%). A score ≥ 5 leads to the most balanced statistical measures, with a sensitivity of 61% and specificity of 62%, and only 1.26 HF patients are needed to be tested to identify a case of low Se status (Table 2). In comparison, were all patients screened for low selenium concentrations, this would result in 1 positive case for every 2.4 patients.

### 3.3. Prevalence of Se, Iron, and Anemia in Association with the Identified Clinical Predictors

**Figure 2:**
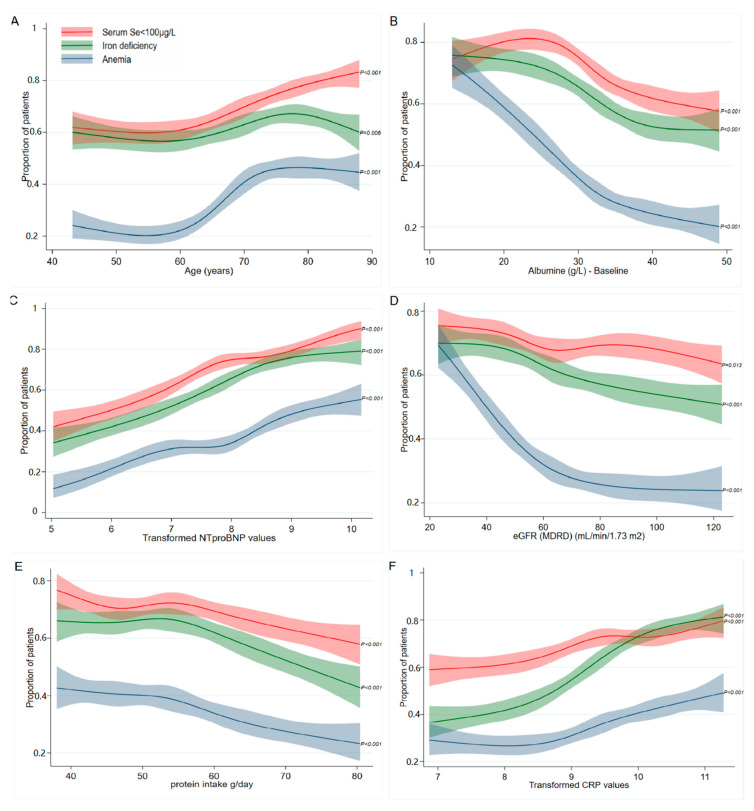
Comparing the prevalence of low serum Se concentrations, ID, and anemia. Restricted cubic splines of the association between the prevalence of serum selenium <100 µg/L, iron deficiency, and anemia and (**A**) age, (**B**) albumin levels, (**C**) transformed NT-proBNP values, (**D**) kidney function (according to the MDRD formula), (**E**) estimated protein intake, and (**F**) transformed CRP values. The solid lines indicate estimates of the prevalence of low selenium concentrations (red), ID (green), and anemia (blue) across continuous levels of the identified clinical determinants of low selenium, fitted using a logistic regression analysis. The shaded areas indicate 95% confidence intervals. The *p*-values indicate the significance of the whole cubic spline model for each factor.

Each predictor for low Se was associated significantly with ID and anemia in restricted cubic splines (*p* < 0.05). Patients aged > 65 years and/or with an albumin <± 32 g/L had a higher risk of having any of these deficiencies (Figure 2A,B). Low Se and ID show similar associations with the NT-proBNP levels and eGFR. Anemia is especially strongly associated with renal function; a sharp increase in the prevalence of anemia can be observed at patients with an eGFR < 60 mL/min/1.73 m^2^ (Figure 2D). Restricted cubic splines were also constructed for the daily protein intake and CRP, as it was shown to be a risk factor for ID [9]. Low Se, ID, and anemia all show a similar increasing prevalence with a lower estimated protein intake (Figure 2E), while CRP was strongly associated with mainly ID (Figure 2F).

### 3.4. Associations with Prognosis Comparing Low Serum Se Concentrations and ID

**Figure 3:**
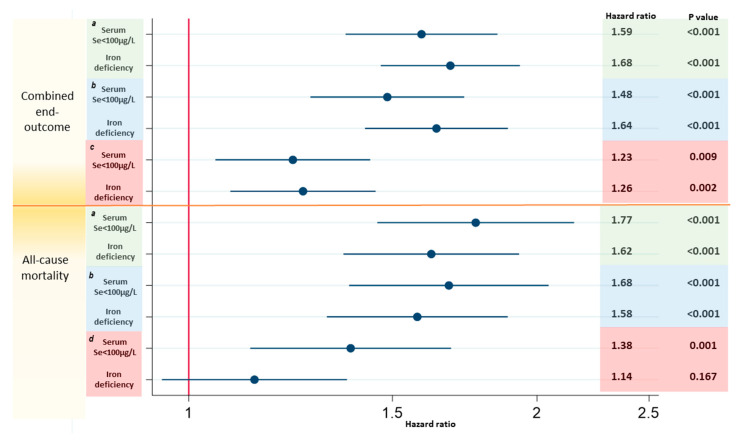
Comparing associations with prognosis for low serum Se concentrations and ID. Cox regression models for the prediction of the combined outcome and all-cause mortality. (a): serum selenium <100 µg/L or iron deficiency as univariate predictors in different models. (b): serum selenium <100 µg/L and iron deficiency together in one model. (c): serum selenium <100 µg/L and iron deficiency together in one model and corrected for the BIOSTAT-CHF risk-model, consisting of age, Heart Failure hospitalization in the year before inclusion, the presence of edema, higher NTproBNP, lower systolic blood pressure, hemoglobin, high-density lipoprotein (HDL) levels, serum sodium concentration, and failure to prescribe a beta-blocker. (d): serum selenium <100 µg/L and iron deficiency together in one model and corrected for the BIOSTAT-CHF mortality risk-model, consisting of age, higher blood urea nitrogen (BUN), NT-proBNP, hemoglobin, and failure to prescribe a beta-blocker.

Both low Se and ID were associated with an unfavorable prognosis univariably and were also independent from each other (Figure 3). In order to assess the associations of low Se and ID with a prognosis independent from each other and known cardiovascular risk factors, we analyzed both by adding them as factors in the previously established BIOSTAT models [20]. Both low Se and ID were significantly associated with the combined 2-year endpoint of HF hospitalizations and mortality (hazard ratio (HR) 1.23; 95% confidence interval (CI) 1.05–1.44; *p* = 0.009 and HR 1.26; 95% CI 1.09–1.45; *p* = 0.002, respectively). Low Se remained independently associated with 2-year all-cause mortality (HR 1.38; 95% CI 1.13–1.69; *p* = 0.001) when corrected for ID (Figure 3).

## 4. Discussion

Using an established cohort of patients with worsening HF, we used independent predictors of low Se (<100 μg/L)—older age, lower levels of albumin, worse kidney function, higher levels of NT-proBNP, and the presence of orthopnea and ID—to establish a prediction tool for low selenium in HF patients. Low serum selenium concentrations were present in 1633 (70.1%) HF patients. By taking a score of 6 or higher in this scoring system, the number of samples needed to be tested for one positive case could be reduced from 2.4 to 1.19. With the exception of CRP, the prevalence of low Se and ID showed similar patterns in relation to the clinical predictors. Additionally, a low Se was independent of ID, and the previously established risk factors were associated with the primary (combined) endpoint and mortality alone.

In contrast to what has been reported previously [24,25], smoking, alcohol, or BMI were not significantly associated with selenium status. Although subjects from most countries had similar selenium levels and geographical location was not independently associated with low selenium levels, relatively high selenium levels were found in subjects from Italy and low levels were seen in subjects from Slovenia (Table A5). Italy was the only country with mean selenium levels of above 100 μg/L, which might be attributed to the Mediterranean diet, which contains selenium-rich components such as fish and nuts [26].

### 4.1. Using Clinical Variables to Predict Low Se

The developed a risk-model that could facilitate the efficient identification of patients at high risk of having a low Se level—for example, in the case of screening for a clinical trial targeting Se as an interventional therapy. With a score of 6 points or higher, approximately 80% of patients with a serum Se < 100 µg/L could be identified. We recently showed that serum Se levels between 70 and 100 µg/L appeared to have similar adverse associations such as deficiency (<70 μg/L), suggesting that values < 100 µg/L might be considered insufficient for the physiological requirement and thus abnormal [27,28]. This is supported by molecular evidence that shows that a serum Se of at least 100 µg/L is needed to have optimal glutathione peroxidase (GPx) and Selenoprotein P activity [29,30,31,32,33]. Determining the Se baseline concentrations is essential, as only those with suboptimal levels might benefit from supplementation, although such a hypothesis needs to be evaluated in a well-designed clinical trial. While the risk-model can help to detect approximately 84% of HF patients with low Se with a score ≥ 6, it should be noted that ruling out low Se is challenging. Even in the population with a score of 0, 43% of the patients had a low Se level.

### 4.2. Clinical Predictors of Low Se

#### 4.2.1. Age

Age has been reported before as an important determinant of Se concentrations [25,34]. A decrease in the serum Se levels was mainly observed in patients older than 70 years. [25,35]. Decreased absorption capacity and impaired intestinal function with aging, as well as increased oxidative stress and inflammation in the elderly, are suggested as underlying causes [36,37]. Dietary intake also seems to play a relevant role, as those with a lower protein intake were more likely to have low Se status, although it was not an independent predictor of Se status (Figure 2E).

#### 4.2.2. NT-proBNP and Orthopnea

In a placebo controlled intervention study, Se supplementation (in combination with coenzyme Q10) showed a reduction in the 10-year cardiovascular mortality and NT-proBNP levels in healthy elderly, supporting the hypothesis of a causal relationship between Se and NT-proBNP [38]. Orthopnea, a sign of congestive HF, represents the deteriorated cardiac health status.

#### 4.2.3. Kidney Function and Albumin

Worse kidney function and lower Se status frequently occur simultaneously [39,40]. A recent in vivo study illustrated that a Se-deficient diet led to significant increase in the urinary protein/creatinine ratio, a reduction in the mitochondrial protein levels, increased oxidative stress markers, as well as histological changes in kidney tissues, suggesting a causative role of Se deficiency in inducing kidney injury [41].

Next to this, several observational patient studies showed a positive correlation between Se and albumin levels [42,43], which was substantiated by in vivo evidence showing that Se supplementation increased albumin concentration [44]. However, residual confounding or a reverse association (i.e., low albumin leads to low Se levels) cannot be excluded, as approximately 10% of the total serum Se binds to albumin [42].

#### 4.2.4. Iron Deficiency

ID as a determinant for low serum Se concentrations has not been reported previously. However, several studies reported the presence of ID in Se-deficient patients [45,46]. Shared risk factors and dietary effects are likely to play a role, although the availability of Se in the diet is more geographically dependent on its concentration in the soil. While they are both mainly absorbed in the duodenum, it is not clear whether there is direct or indirect physiological interaction between both elements.

### 4.3. The Prevalence of Low Serum Se Concentrations, ID, and Anemia in Relation to the Determinants

Our data show that low Se and ID have important similarities in their associations with patient characteristics. The similarity in the patterns supports the hypothesis that both elements share similar underlying detrimental processes. We previously reported that inflammation, chronic kidney disease, lower hemoglobin levels, and lower serum albumin levels were independent predictors for ID in HF patients [9].

### 4.4. Low Serum Se Concentrations Associate with Worse Prognosis than ID

Including both ID and low Se concentration in the BIOSTAT prediction models indicated that low Se status is independently associated with mortality in HF patients when additionally corrected for ID (Figure 3). Clinical trials of intravenous iron supplements have shown improvements in the symptoms and well-being of HF patients, and trials investigating the effects on morbidity and mortality are underway. Unlike iron, selenium is readily absorbed orally. However, it should be acknowledged that, although we showed that low Se was associated with a similar or worse prognosis compared to ID, more evidence from well-designed clinical trials, like those performed for ID, is needed to evaluate the potential benefits for Se supplementation in HF.

### 4.5. Strengths and Limitations

The BIOSTAT-CHF cohort is a large HF cohort in which comprehensive clinical and biochemical markers were measured. Nevertheless, it should be acknowledged that it included mainly patients with Caucasian origins. The development of a clinical tool to predict Se levels using clinical factors remains as a challenging task, as the sensitivity of having a score 6 in our model is 44%. This could be attributed to the high prevalence of low Se levels in this cohort, and this may indicate that determining the Se status in patients with HF may be standardized when the clinical benefits are established in clinical trials. Additionally, due to the observational nature of the study, establishing a causal relationship between our results and Se concentrations is not possible. No data were currently available on other markers of selenium-dependent pathways, nor oxidative stress. As a result, other signaling components such as Selenoprotein P or gluthatione peroxidases were not investigated next to the serum selenium.

## 5. Conclusions

In this study, we developed a prediction tool which identifies HF patients at higher risk of having a low Se level. Yet, it remains a challenge to exclude patients with low Se levels based on clinical characteristics alone. Low Se and ID share to large extent similar predictors. Finally, a lower Se status is associated with similar or worse prognosis compared to, and independent of, ID.

## Figures and Tables

**Table 1:** Independent determinants of low selenium concentrations.

Factor	Odds Ratio	Standard Error	z	*p* Value	95% Confidence Interval	
eGFR (Kidney Function)	1.01	0.00	2.68	0.007	1.00	1.01
Iron Deficiency	1.36	0.14	3.02	0.002	1.11	1.66
NTproBNP	1.30	0.04	8.71	<0.001	1.23	1.39
Albumin	0.97	0.01	−4.55	<0.001	0.96	0.99
Orthopnoea	1.43	0.15	3.31	0.001	1.16	1.77
Age	1.02	0.00	4.63	<0.001	1.01	1.03

**Table 2:** Statistical measures of all scores.

Score	ROC ^#^	Sensitivity	Specificity	NPV ^‡^	PPV ^†^	NNT *
1	0.51	99%	2%	57	70	1.42
2	0.53	96%	9%	51	71	1.40
3	0.57	89%	24%	49	73	1.36
4	0.60	77%	42%	44	76	1.32
5	0.62	61%	62%	41	79	1.26
6	0.62	44%	80%	38	84	1.19
7	0.57	25%	89%	34	85	1.18
8	0.54	12%	96%	32	88	1.14
9	0.51	3%	99%	30	88	1.13
10	0.50	1%	100%	30	100	1

^#^ Receiver operating characteristic; ^‡^ Negative Predictive Value; ^†^ Positive Predictive Value; * number needed to treat.

## Abbreviations

HF, heart failure; CI, confidence interval; CRP, C-reactive protein; GPx, glutathione peroxidase; HFrEF, heart failure with reduced ejection fraction; HR, hazard ratio; ID, iron deficiency; LVEF, left ventricle ejection fraction; MDRD, modification of diet in renal disease; NT-proBNP, N-terminal pro-B-type natriuretic peptide; OR, odds ratio; Se, selenium; TSAT, transferrin saturation; eGFR, estimated Glomerular Filtration Rate.

## Appendix A

**Table A1:** Selected variables that entered the analysis and their degree of missingness.

Factor		Percentage
Age (years)	0	0.00
Females (%)	0	0.00
Estimated protein intake (g/day)	167	7.17
NYHA functional class	288	12.37
Orthopnoea	5	0.21
Iron deficiency	13	0.55
Atrial fibrillation	0	0.00
CRP (mg/L)	98	4.21
NTproBNP (ng/L)	58	2.49
eGFR (MDRD) (mL/min/1.73 m2)	7	0.30
Albumine (g/L)	12	0.52
Hemoglobin	200	8.59
Beta-blockers - baseline	0	0.00
Aldosteron antagonist	0	0.00
Country	0	0.00
Heart rate (b.p.m)	6	0.99

**Table A2:** Baseline characteristics.

Factor	Level	Value	Se ≥ 100 µg/L	Se < 100 μg/L	*p*-Value
N		2328	695	1633	
Selenium (µg/L)		89.1 (24.8)	118.5 (18.2)	76.6 (14.5)	<0.001
Demographics					
Age (years)		68.8 (12.0)	66.0 (11.8)	70.1 (11.9)	<0.001
Females (%)		607 (26.1%)	150 (21.6%)	457 (28.0%)	0.001
BMI (kg/m2)		27.2 (24.1, 30.6)	27.7 (24.4, 30.7)	26.9 (24.0, 30.6)	0.072
Estimated protein intake (g/day)		53.4 (46.5, 62.0)	54.6 (47.3, 64.6)	52.9 (46.1, 61.1)	<0.001
Ischemic etiology		1057 (46.2%)	315 (46.2%)	742 (46.2%)	0.98
LVEF (%)		30.0 (25.0, 36.0)	30.0 (25.0, 35.0)	30.0 (25.0, 38.0)	0.002
HFrEF (LVEF < 40)		1691 (81.3%)	554 (87.1%)	1137 (78.7%)	<0.001
NYHA functional class	Class I	214 (10.5%)	83 (13.3%)	131 (9.3%)	<0.001
	Class II	1077 (52.8%)	353 (56.6%)	724 (51.1%)	
	Class III	672 (32.9%)	174 (27.9%)	498 (35.2%)	
	Class IV	77 (3.8%)	14 (2.2%)	63 (4.4%)	
Previous hospitalization for HF		720 (30.9%)	201 (28.9%)	519 (31.8%)	0.17
Heart rate (b.p.m)		76.0 (66.0, 90.0)	75.0 (65.0, 86.0)	77.0 (68.0, 90.0)	<0.001
Systolic blood pressure (mmHg)		120.0 (110.0, 138.0)	120.0 (110.0, 135.0)	120.0 (110.0, 140.0)	0.39
Diastolic blood pressure (mmHg)		74.9 (13.1)	75.0 (12.3)	74.8 (13.5)	0.71
Peripheral oedema		1149 (59.4%)	255 (44.6%)	894 (65.6%)	<0.001
Pulmonary oedema		507 (32.2%)	128 (30.7%)	379 (32.8%)	0.44
Elevated JVP	No	1025 (62.9%)	393 (75.9%)	632 (56.8%)	<0.001
	Yes	514 (31.5%)	102 (19.7%)	412 (37.1%)	
	Uncertain	91 (5.6%)	23 (4.4%)	68 (6.1%)	
Orthopnoea		802 (34.5%)	180 (25.9%)	622 (38.2%)	<0.001
Hepatomegaly		331 (14.3%)	102 (14.7%)	229 (14.1%)	0.71
Completion of 6MWT		1462 (64.9%)	499 (73.4%)	963 (61.2%)	<0.001
Result 6MWT (m)		330.0 (180.0, 429.0)	365.5 (240.0, 449.0)	310.0 (143.0, 420.0)	<0.001
KCCQ (overall score)		49.0 (31.3, 66.7)	56.3 (38.5, 71.9)	44.8 (29.2, 63.5)	<0.001
Smoking (none, past, current)	None	853 (36.7%)	235 (33.9%)	618 (37.9%)	0.18
	Past	1138 (48.9%)	354 (51.0%)	784 (48.1%)	
	Current	334 (14.4%)	105 (15.1%)	229 (14.0%)	
Current alcohol use		644 (27.7%)	196 (28.3%)	448 (27.5%)	0.69
Medical history					
Myocardial infarction		891 (38.3%)	258 (37.1%)	633 (38.8%)	0.46
CABG		402 (17.3%)	133 (19.1%)	269 (16.5%)	0.12
Valvular surgery		171 (7.3%)	54 (7.8%)	117 (7.2%)	0.61
PCI		512 (22.0%)	162 (23.3%)	350 (21.4%)	0.32
Atrial fibrillation		1048 (45.0%)	272 (39.1%)	776 (47.5%)	<0.001
Device therapy		580 (24.9%)	190 (27.3%)	390 (23.9%)	0.078
Stroke		214 (9.2%)	53 (7.6%)	161 (9.9%)	0.088
Peripheral arterial disease		251 (10.8%)	75 (10.8%)	176 (10.8%)	0.99
Renal insufficiency (eGFR <60 mL/min)		1068 (46.0%)	287 (41.4%)	781 (48.0%)	0.003
Renal disease (history)		646 (27.7%)	167 (24.0%)	479 (29.3%)	0.009
Hypertension		1452 (62.4%)	428 (61.6%)	1024 (62.7%)	0.61
Diabetes mellitus		750 (32.2%)	222 (31.9%)	528 (32.3%)	0.85
COPD		403 (17.3%)	109 (15.7%)	294 (18.0%)	0.18
Current malignancy		87 (3.7%)	18 (2.6%)	69 (4.2%)	0.057
Laboratory					
CRP (mg/L)		13,142.2 (5818.3, 26416.6)	11,091.9 (4127.5, 22689.7)	14,151.5 (6760.5, 27734.3)	<0.001
Creatinin (µmol/L)		101.0 (82.2, 129.0)	100.0 (82.1, 123.8)	101.4 (82.2, 131.6)	0.24
Urea (mmol/L)		11.3 (7.6, 18.2)	12.1 (7.8, 19.2)	11.0 (7.5, 17.8)	0.049
Proteinuria (mg/dL)		5.0 (0.0, 20.0)	5.0 (0.0, 20.0)	2.0 (0.0, 20.0)	0.68
Sodium (mmol/L)		139.2 (3.9)	139.4 (3.5)	139.1 (4.1)	0.25
Potassium (mmol/L)		4.3 (0.6)	4.3 (0.5)	4.3 (0.6)	0.95
NTproBNP (ng/L)		2698.0 (1179.0, 5751.0)	1628.5 (696.5, 3791.0)	3224.0 (1483.0, 6635.5)	<0.001
GDF15, median (IQR)		2720.0 (1712.0, 4556.0)	2133.0 (1346.0, 3577.0)	3021.5 (1915.5, 5224.5)	<0.001
eGFR (MDRD) (mL/min/1.73 m2)		65.0 (26.3)	67.7 (27.5)	63.9 (25.7)	0.001
IL-6 (pg/mL)		5.2 (2.8, 10.2)	3.9 (2.1, 7.4)	5.8 (3.2, 11.4)	<0.001
Total bilirubin (µmol/L)		14.0 (9.9, 21.0)	13.2 (9.1, 18.8)	14.3 (10.0, 22.2)	0.001
Leucocytes (10e9/L)		8.3 (2.9)	8.3 (2.7)	8.3 (2.9)	0.95
Troponin I (µg/L)		0.0 (0.0, 0.1)	0.0 (0.0, 0.1)	0.0 (0.0, 0.1)	0.006
Troponin T (µg/L)		0.0 (0.0, 0.1)	0.0 (0.0, 0.1)	0.0 (0.0, 0.1)	0.71
Glucose (mmol/L)		6.3 (5.4, 7.9)	6.3 (5.4, 8.0)	6.2 (5.3, 7.9)	0.22
HbA1c (%)		6.3 (5.7, 7.1)	6.4 (5.8, 7.3)	6.2 (5.7, 7.0)	0.24
ASAT (U/L)		25.4 (19.0, 35.0)	25.0 (19.0, 35.0)	26.0 (20.0, 35.0)	0.35
ALAT (U/L)		25.0 (17.0, 38.0)	28.0 (20.0, 42.0)	23.0 (16.0, 36.0)	<0.001
γ-GT (U/L)		55.0 (28.0, 108.5)	44.0 (26.0, 84.0)	60.0 (29.0, 118.0)	<0.001
Alkaline phosphatase (µg/L)		84.0 (65.0, 118.0)	77.0 (62.0, 104.0)	88.0 (67.0, 122.0)	<0.001
HDL (mmol/L)		1.1 (0.8, 1.3)	1.1 (0.9, 1.3)	1.0 (0.8, 1.3)	0.065
LDL (mmol/L)		2.6 (1.1)	2.8 (1.1)	2.5 (1.1)	<0.001
Total cholesterol (mmol/L)		4.3 (1.3)	4.5 (1.4)	4.2 (1.3)	<0.001
Triglycerides (mmol/L)		1.2 (0.9, 1.7)	1.3 (1.0, 1.8)	1.2 (0.9, 1.6)	<0.001
TSH (mU/L)		1.8 (1.2, 3.0)	1.8 (1.1, 3.2)	1.8 (1.2, 3.0)	0.85
FT4 (pmol/L)		16.8 (7.6)	17.3 (10.6)	16.5 (5.6)	0.29
Albumine (g/L)		33.0 (27.0, 38.0)	34.0 (30.0, 40.0)	32.0 (26.5, 37.0)	<0.001
Hemoglobin (g/dL)		13.2 (1.9)	13.6 (1.8)	13.0 (1.9)	<0.001
Anemia		772 (36.3%)	176 (28.9%)	596 (39.2%)	<0.001
Mean corpuscular volume (fL)		90.4 (8.6)	91.0 (7.8)	90.2 (8.9)	0.097
Iron deficiency		1436 (62.0%)	357 (51.7%)	1079 (66.4%)	<0.001
Iron (mg/dL)		44.7 (27.9, 72.6)	55.9 (39.1, 78.2)	44.7 (27.9, 67.0)	<0.001
Ferritin (μg/L)		101.0 (50.0, 191.5)	126.0 (62.5, 208.0)	93.0 (46.0, 185.0)	<0.001
Transferrin (mg/dL)		206.0 (69.7)	211.9 (67.9)	203.5 (70.4)	0.008
Transferrin saturation (%)		17.1 (10.9, 24.9)	19.9 (13.3, 27.2)	15.9 (9.9, 23.5)	<0.001
Hepcidin (nmol/L)		6.3 (2.2, 16.5)	7.5 (3.1, 17.3)	5.7 (1.8, 15.9)	<0.001
sTfR (mg/L)		1.8 (1.0)	1.5 (0.8)	1.9 (1.1)	<0.001
Medication					
Betablocker - baseline		1941 (83.4%)	597 (85.9%)	1344 (82.3%)	0.033
ACE/ARB - baseline		1685 (72.4%)	522 (75.1%)	1163 (71.2%)	0.055
Antiplatelets		1208 (51.9%)	363 (52.2%)	845 (51.7%)	0.83
P2Y12V2		359 (15.4%)	101 (14.5%)	258 (15.8%)	0.44
Diuretics		2326 (99.9%)	694 (99.9%)	1632 (99.9%)	0.53
Loop Diuretics		2317 (99.5%)	690 (99.3%)	1627 (99.6%)	0.26
Oral anti-diabetics		469 (62.5%)	133 (59.9%)	336 (63.6%)	0.34
Insuline use		301 (40.1%)	93 (41.9%)	208 (39.4%)	0.52
Proton pump inhibitor		815 (35.0%)	237 (34.1%)	578 (35.4%)	0.55
Acenocoumarol		896 (38.5%)	258 (37.1%)	638 (39.1%)	0.38
Aldosteron antagonist		1241 (53.3%)	398 (57.3%)	843 (51.6%)	0.013
Spironolactone		855 (36.7%)	250 (36.0%)	605 (37.0%)	0.62

LVEF, Left Ventricular Ejection Fraction; HF, Heart Failure; JVP, Jugular Venous Pressure; CABG, Coronary Artery Bypass Grafting; PCI, Percutaneous coronary intervention; COPD, Chronic Obstructive Pulmanory Disease.

**Table A3:** Results of the bootstrapping analysis.

	N. of Repetion
Age (years)	856
NTproBNP (per doubling)	1000
eGFR (MDRD) (mL/min/1.73 m2)	838
Albumine (g/L)	859
Iron deficiency	553
Orthopnoea	813

**Table A4:** Results of LASSO penalized regression.

Predictor ^#^	Penalized Odds Ratios
NTproBNP	1.32
Albumin	0.98
Kidney function (eGFR MDRD)	1.01
Age	1.01
Iron deficiency	1.23
Orthopnoea	1.38

^#^ Variables are ordered based on their absolute values of their coefficients, starting from the largest. LASSO, Least Absolute Shrinkage and Selection Operator.

**Table A5:** Selenium values based on geographical location.

Country	Number of Included Patients	Mean Selenium	Percentage of Patients < 100 μg/L
the Netherlands	386	86.00	76.68
France	243	80.70	80.66
Germancy	87	90.69	67.82
Serbia	382	91.23	66.49
Slovenia	43	66.83	97.67
Greece	285	90.87	68.42
Italy	297	115.48	27.61
Norway	108	79.16	86.11
Sweden	97	79.49	84.54
Poland	239	79.33	87.45
United Kingdom	161	84.42	77.64

**Table A6:** Number of patients with selenium <100 μg/L per cluster per variable.

Factor	0 Point	1 Point	2 Points	Total Number of Patients
NTproBNP	555	352	685	1592
eGFR Kidney Function	343	977	307	1627
Albumin	264	1050	310	1624
Age	374	972	287	1633
Iron deficiency	545	1079	NA	1624
Orthopnoea	1006	622	NA	1628
